# Using crystallography tools to improve vaccine formulations

**DOI:** 10.1107/S205225252101071X

**Published:** 2021-11-25

**Authors:** Márcia Carvalho de Abreu Fantini, Cristiano Luis Pinto Oliveira, José Luiz de Souza Lopes, Tereza da Silva Martins, Milena Apetito Akamatsu, Aryene Góes Trezena, Milene Tino-De- Franco, Viviane Fongaro Botosso, Osvaldo Augusto Brazil Esteves Sant’Anna, Nikolay Kardjilov, Martin Kjaerulf Rasmussen, Heloísa Nunes Bordallo

**Affiliations:** a Physics Institute, University of São Paulo, Rua do Matão 1371, São Paulo, SP 05508-090, Brazil; b Chemistry Department, Federal University of São Paulo, Rua São Nicolau, 210, 2o andar, Diadema, SP 09913-030, Brazil; c Bioindustrial Center, Butantan Institute, Avenida Vital Brazil, 1500, São Paulo, SP 05503-900, Brazil; d Immunogenetic Laboratory, Butantan Institute, Avenida Vital Brazil, 1500, São Paulo, SP 05503-900, Brazil; e Virology Laboratory, Butantan Institute, Avenida Vital Brazil, 1500, São Paulo, SP 05503-900, Brazil; f Immunochemistry Laboratory, Butantan Institute, Avenida Vital Brazil, 1500, São Paulo, SP 05503-900, Brazil; g HZB für Materialien und Energie, Helmholtz-Zentrum Berlin, Hahn-Meitner-Platz 1, 14109 Berlin, Germany; h Niels Bohr Institute, University of Copenhagen, Universitetsparken 5, Copenhagen 2100, Denmark

**Keywords:** oral vaccines, porous silica, SAXS, XAS, imaging

## Abstract

This article summarizes developments attained in oral vaccine formulations based on the encapsulation of antigen proteins inside porous silica matrices. These vaccine vehicles show great efficacy in protecting the proteins from the harsh acidic stomach medium, allowing the Peyer’s patches in the small intestine to be reached and consequently enhancing immunity. Focusing on the pioneering research conducted at the Butantan Institute in Brazil, the optimization of the antigen encapsulation yield is reported, as well as their distribution inside the meso- and macroporous network of the porous silica. As the development of vaccines requires proper inclusion of antigens in the antibody cells, X-ray crystallography is one of the most commonly used techniques to unveil the structure of antibody-combining sites with protein antigens. Thus structural characterization and modelling of pure antigen structures, showing different dimensions, as well as their complexes, such as silica with encapsulated hepatitis B virus-like particles and diphtheria anatoxin, were performed using small-angle X-ray scattering, X-ray absorption spectroscopy, X-ray phase contrast tomography, and neutron and X-ray imaging. By combining crystallography with dynamic light scattering and transmission electron microscopy, a clearer picture of the proposed vaccine complexes is shown. Additionally, the stability of the immunogenic complex at different pH values and temperatures was checked and the efficacy of the proposed oral immunogenic complex was demonstrated. The latter was obtained by comparing the antibodies in mice with variable high and low antibody responses.

## Abstract 2

A review is presented on the strategic use of scattering and imaging tools to design immunologic complexes based on porous silica for oral vaccine formulations.

## 1. Introduction

Subcutaneous vaccine administration is the most used protocol in immunization campaigns due to its highest degree of effectiveness. However, it is well known that more than 90% of the pathogens enter the body by mucosal sites. Consequently, developing mucosal vaccines that act as an extra barrier against pathogen invasion is an interesting idea (Miquel-Clopés *et al.*, 2019 ▸; Jiang *et al.*, 2019 ▸). Even though the administration of oral and nasal vaccination seems to be the most natural way, there is a major challenge that needs to be resolved: antigens must safely overcome the body’s barriers to generate antibodies and induce immunization (Goffin *et al.*, 2019 ▸; Coria *et al.*, 2019 ▸; Miao *et al.*, 2019 ▸). This means that efficient and biosafe carriers must be developed (Zhang *et al.*, 2015 ▸). Owing to advances in nanotechnology over the last two decades, not only has our ability to control the synthesis of new bionano materials been improved, but also new nano-tools to scrutinize the nano-world, in particular, related to immunology (Rosales-Mendoza & González-Ortega, 2019 ▸) have been developed. On one hand the achievements of nanotechnology should be celebrated; however, the safety of bionano-products is an important concern for human use, including immune-toxicity (Dobrovolskaia *et al.*, 2009 ▸; Liangjiao *et al.*, 2019 ▸). Thus, many adjuvants or protective carrier vehicles have been tested. Nowadays, those certified are mostly composed of polymers, liposomes, aluminium hydroxide as well as synthetic mesoporous silica (Mirchamsy *et al.*, 1997 ▸; Calderón & Sosnik, 2015 ▸; Mercuri *et al.*, 2006 ▸; Vallhov *et al.*, 2007 ▸). The improvement triggered by silica on the immunological responsiveness has been known since 1987 (Gennari *et al.*, 1987 ▸). However, as studies on silica nanoparticles toxicity are controversial (Kupferschmidt *et al.*, 2013 ▸; Chen *et al.*, 2018 ▸, 2019 ▸), the use of this powerful protagonist adjuvant for new vaccine developments faces some challenges. The main concerns being the decrease of immunization when micrometric particles are used, since the administration is not continuous, and the fact that silica can be easily excreted (He *et al.*, 2011 ▸; Mariano-Neto *et al.*, 2014 ▸; Scaramuzzi *et al.*, 2016 ▸). Despite such concerns, from 2006 to 2020 more than fifty papers, including extensive reviews (Mody *et al.*, 2013 ▸; Zhao *et al.*, 2014 ▸), reported on how these nanostructured porous materials are powerful candidates for new and optimized oral vaccines.

Here we discuss our own pioneer insight (International Patent WO07/030901) on the use of ordered mesoporous silica (OMS), SBA-15 type (Zhao *et al.*, 1998 ▸) as adjuvant, as well as a protective vehicle for antigens in its form of porous matrix. In this review article we show how SBA-15 is able to protect different antigens while inducing higher immunological response. Another important issue is the stability of the antigens encapsulated while exposed to rush environments, such as temperature and pH variations (Lopes *et al.*, 2019 ▸). Throughout the manuscript we must recall that to achieve an effective vaccine it is necessary to distribute the antigens homogeneously inside their vehicles, without aggregation, such that the T-cells can reach all of them, allowing immunity to be achieved. Thus, one purpose of our work was to answer if non-agglomerated hepatitis B surface antigen (HBsAg) is distributed uniformly in SBA-15 and whether HBsAg is indeed protected by attaching to the openings of the mesopores. This was done by combining X-ray and neutron tomography experiments, which allowed determination of the ratio HBsAg:SBA-15 with the least agglomeration of vaccine. Our second goal was to conduct measurements on the diphtheria anatoxin in SBA-15 vaccine. The interest comes from the fact that diphtheria anatoxin (dANA) is much smaller, with a maximum length around 9 nm (Rasmussen *et al.*, 2021 ▸), than HBsAg, a globular particle around 30 nm (Scaramuzzi *et al.*, 2016 ▸), making dANA an excellent candidate for full encapsulation into SBA-15, as in principle it is small enough to enter the mesopores, having a mean mesopore diameter of around 10 nm.

## 2. Experimental

### 2.1. Silica synthesis and antigen encapsulation

**Figure 1:**
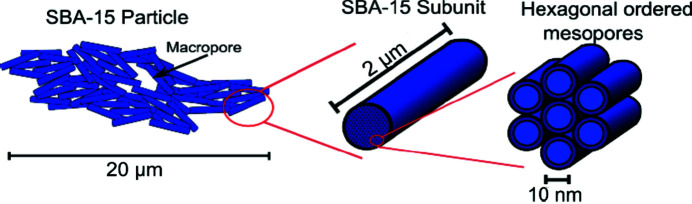
Sketch of SBA-15 showing the macropores in the 20 µm particle, the 2 µm rod-shaped subunit and the 10 nm hexagonal-ordered mesopores [reproduced from the work by Rasmussen *et al.* (2017 ▸)].

Fig. 1 ▸ depicts a sketch of the OMS mesoscopic parameters, micropores less than 2 nm in the amorphous silica walls; ordered mesopores (diameter ∼10 nm), organized in a bidimensional hexagonal *p*6*mm* structure; and macropores larger than 50 nm between the silica particles, whose dimensions reach 20 µm.

The SBA-15 matrix used here was prepared according to the synthesis detailed in previous work (Mariano-Neto *et al.*, 2014 ▸). Antigen encapsulation was performed by mixing the protein dissolved in phosphate buffer saline (PBS) solution (pH = 7.4) with silica powder. This physical mixture was first stirred for a few minutes, followed by a drying process at 35 ± 5°C at rest in air. Different antigen:silica mass ratios (for example, 1:10, 10 mg of HBsAg and 100 mg of SBA-15) were analysed to evaluate the encapsulation yield. Two sources of SBA-15 were analysed, one produced on an industrial prototype scale (around 40 g, named L1) and the other synthesized on a small laboratory scale (maximum of 4 g, named L2). Even though the synthesis of the SBA-15 type of OMS seems to be a very straightforward process, our experience showed that strict control of all synthesis parameters, such as temperature, stirring speed, humidity, evaporation, *etc.* is mandatory to produce reproducible textural, structural and morphological properties.

### 2.2. Materials characterization

The analysis of pristine SBA-15, SBA-15 plus PBS and SBA-15 encapsulated with the antigens and PBS was performed by associating nitro­gen adsorption isotherms (NAIs) with X-ray and neutron scattering methods. This approach provided a complete description of the structural and morphological properties of the immunogenic complexes. Our previous works explained in detail how the complementary techniques, such as NAI, scanning electron microscopy (SEM) with energy-dispersive X-ray spectroscopy (EDS) and transmission electron microscopy (TEM), dynamic light scattering (DLS), thermogravimetric analysis (TGA), synchrotron radiation circular dichroism (SRCD) and intrinsic tryptophan fluorescence spectroscopies, were used to further evaluate the effectiveness of our strategies to develop oral vaccines, including biological assays. Thus, these results will not be detailed here, we refer the reader to the literature (Carvalho *et al.*, 2010 ▸; Mariano-Neto *et al.*, 2014 ▸; Scaramuzzi *et al.*, 2016 ▸; Garcia *et al.*, 2016 ▸; Lopes *et al.*, 2019 ▸; Mercuri *et al.*, 2006 ▸; Rasmussen *et al.*, 2019 ▸ *b*, 2021 ▸). These applications will be described and correlated in the Discussion[Sec sec3].

#### 2.2.1. Nitro­gen adsorption isotherm

The NAI provides the surface area and pore-size distribution. Data were collected by an ASAP 2020 porosimeter, using nitro­gen gas. Degassing of the samples at 40°C was carried out before the isotherm measurements.

#### 2.2.2. Small-angle X-ray scattering

Small-angle X-ray scattering (SAXS) measurements were performed using a Nanostar camera (Bruker) with a Xenocs microfocus source and a bidimensional Vantec2000 detector or a Xeuss camera (Xenocs) with a solid-state Pilatus detector. Data were corrected by detector response, transmittance of the samples and parasitic scattering, such that the absolute SAXS intensity was calculated, using pure water as reference (Oliveira, 2011 ▸). The scattering vector *q* = (4πsinθ)/λ was taken from 0.1 to 3.5 nm^−1^, with Cu *K*α radiation, λ = 0.15418 nm. *In situ* SAXS experiments were performed to follow the release of the antigens from SBA-15. A mass of 40 mg SBA-15 powder was dissolved in 45 ml of different solutions either in neutral pH of 7.4 or mimicking the gastric (pH 1.2) and intestinal (pH 6.8) environments under continuous magnetic stirring. The liquid was then pumped with a constant flow through a capillary in front the X-ray beam while frames of 2 min were taken. *In situ* SAXS experiments of SBA-15 with the encapsulated vaccines were performed in order to understand the release behaviour from SBA-15.

The samples analysed by *in situ* SAXS experiments are described in Table 1 ▸. The sample labelled 1:30(HBsAg) plus Eudragit was coated with Eudragit, which is a polymer that disintegrates when the pH is higher than 6.2. Eudragit can protect the encapsulated HBsAg from the gastric acid (pH 1.2) and disintegrate when entering the intestine (pH 6.8), to allow the release of the vaccine at the target area. The Eudragit coating was made by mixing the sample 1:30(HBsAg) with this polymer in a mass relation of 1:1, allowing this mixture to dry at 35°C. SAXS experiments were performed with both SBA-15 silica sources. As mentioned before, the first batch (L1) was synthesized in an industrial prototype plant, where the synthesis of SBA-15 was scaled up towards industrial production, while the second batch (L2) was synthesized in a small laboratory setup. The *in situ* SAXS experiments of HBsAg were performed with both L1 and L2 types of SBA-15. Experiments with dANA were performed with L2 silica. Details of the experiments are described in Table 1 ▸.

The *in situ* SAXS data were registered over 12 h for pure SBA-15 (L1 and L2) and for 48 h for the samples containing the antigens.

#### 2.2.3. Neutron and X-ray attenuation tomography

Neutron attenuation tomography was performed using the CONRAD instrument at the Helmholtz Zentrum Berlin (HZB), Germany, which is equipped with a 2k × 2k CCD neutron camera. X-ray measurements were carried out on a 150 kV µC T X-ray tube with a 2048 × 2048 pixel amorphous-Si flat-panel detector also at HZB. For direct comparison, data were collected on the same powder samples, mounted in aluminium rings with a diameter of 1 cm and a height of 0.4 cm, with the same pixel size of 6.37 µm.

#### 2.2.4. X-ray phase contrast tomography

X-ray phase contrast tomography (XPCT) was performed using the ID16B Nanoanalysis beamline at the European Synchrotron Radiation Facility (ESRF), France. Powder samples were mounted in capillaries with a radius of 400 nm and data were collected using a monochromatic beam with a photon energy of 17 keV. A Frelon 4M camera was used to make 3200 projections for each sample. Measurements were taken to reconstruct tomograms with pixel sizes of 25, 50 and 100 nm. In order to properly determine the phase shift, the measurements were performed with four different sample–detector distances for each sample. The tomograms were reconstructed using the software package provided by the beamline.

#### 2.2.5. X-ray absorption spectroscopy

Scanning transmission X-ray microscopy (STXM) and near-edge X-ray absorption fine structure (NEXAFS) experiments were carried out at the PolLux beamline at the Swiss Light Source (SLS) at the Paul Scherrer Institute (PSI), Switzerland. For all samples, STXM images were acquired with 390 eV energies just above the carbon *K*-edge. The images were made with a field of view between 5 × 5 µm and 30 × 30 µm and a spatial resolution of 200 nm. NEXAFS spectra were also measured for the particles around the carbon *K*-edge in the energy range 270–350 eV in 355 steps. After inspection of the NEXAFS spectra, the energies of interest were selected and STXM images were collected around the carbon edge using 287.1 and 288.1 eV.

## 3. Results and discussion

**Figure 2:**
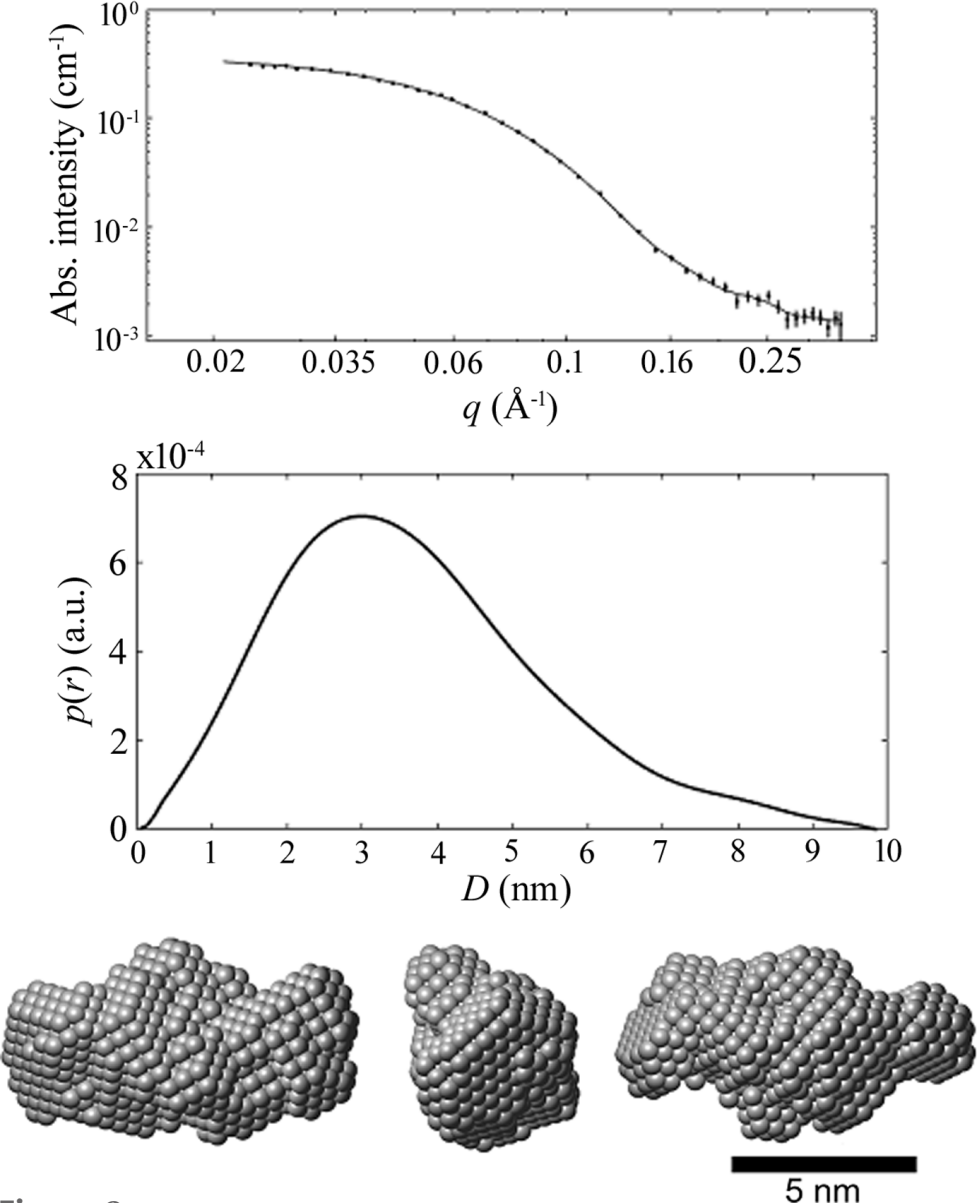
dANA SAXS results in PBS together with its corresponding PDF, *p*(*r*) and particle form obtained from a simulation model [reproduced from the work by Rasmussen *et al.* (2017 ▸)].

**Figure 3:**
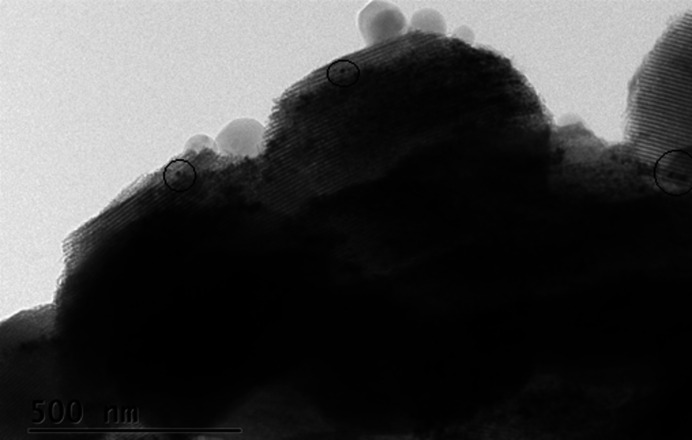
TEM image of dANA inside SBA-15 (L2) mesopores [reproduced from the work by Rasmussen *et al.* (2021 ▸)].

SAXS characterization of the pure antigens in PBS solution (pH = 7.4 and *T* = 25°C) showed that HBsAg is a globular particle with mean diameter of 30 ± 5 nm, hence larger than the silica mean pore diameter *D* = 10.4 ± 0.6 nm, confirmed by DLS and TEM images (Rasmussen *et al.*, 2019 ▸ *a*). Therefore, the HBsAg particles are in the macroporous region of SBA-15, and may also close the entrance of the mesopore and cluster in PBS. dANA, on the other hand, has dimensions similar to the mean diameter of the mesopore, taken from simulation of the SAXS results shown in Fig. 2 ▸ (Rasmussen *et al.*, 2021 ▸), implying that the dANA is protected inside the mesopores, as showed by DLS and TEM in Fig. 3 ▸ (Rasmussen *et al.*, 2021 ▸). Table 2 ▸ presents the NAI results of HBsAg and dANA inside SBA-15 (L2) for different mass ratios, confirming the higher encapsulation of the later.

The SAXS scattering intensity results *I*(*q*) were simulated according to the literature (Mariano-Neto *et al.*, 2014 ▸; Garcia *et al.*, 2016 ▸), given by

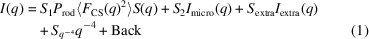

The first term in Equation (1) describes the scattering contribution from the hexagonal ordered mesopore structure, where *S*_1_ is a scale factor, *P*_rod_〈*F*_CS_(*q*)^2^〉 is the particle form factor and *S*(*q*) is the structure factor. The second term designates the scattering from the micropores, *S*_2_ being a scale factor and *I*_micro_(*q*) their intensity. The third term is the contribution to the scattering from ellipsoids used to model unspecific aggregates in the system. Finally, the fourth term is a *q* ^−4^-dependent contribution used to approximate Porod contribution from the hexagonal mesopore structure (initial part of the curve at low angles) and the fifth term is a constant background to account for some remaining contribution of the solvent (Pedersen, 1997 ▸). Table 3 ▸ describes the fitting parameters used in the *I*(*q*) equation.

Smearing from experimental effects, originating from the finite width of the X-ray beam and detector resolution, were also computed. For the setup used in this work, the resolution function can be described by a Gaussian distribution based on the beam width at the detector using a *R*(<*q*>, *q*) function, describing the probed *q*-values for the scattering vector <*q*> (Sundblom *et al.*, 2009 ▸):

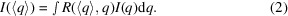

**Figure 4:**
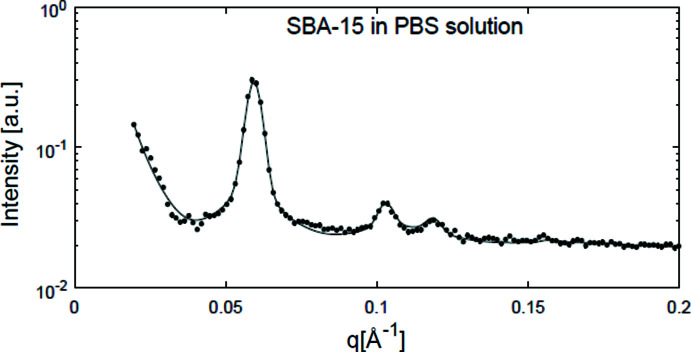
SAXS data for SBA-15 (L1) in PBS solution averaged over 1 h of measurement (points) and the corresponding fitting (continuous line).

First, the simple case of the pure SBA-15 (L1) dispersed in the PBS solution was investigated. The scattering obtained for pure SBA-15 dispersed in PBS solution was averaged over 1 h of measurement and fitted using a value for *S_*q*−4_* different from zero. The result is given in Fig. 4 ▸. To better track the parameters of this process as a function of time, the data were then averaged over 20 intervals every 30 min and fitted with the same model. Analysis of two parameters, ρ and *S*_extra_, where ρ is the ratio of the electron density of the silica pore wall divided by the electron density inside the mesopore, normalized to their initial value, showed a decrease of ρ with time, due to the electron density increasing in the mesopore. This observation is a result of the salt entering the mesopore immediately after SBA-15 is dispersed into the PBS solution. On the other hand, *S*_extra_ remained null during the measurement time, showing that the salt does not agglomerate outside the mesopores of SBA-15 while in solution.

**Figure 5:**
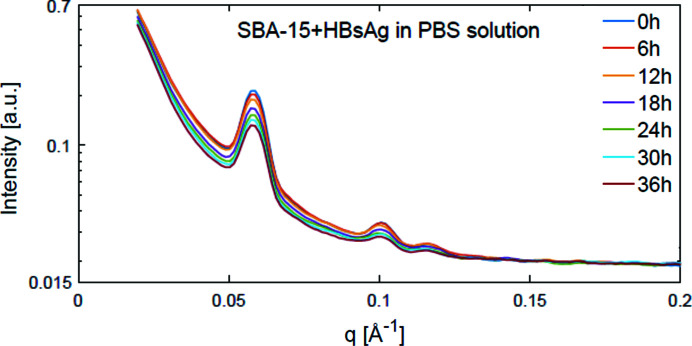
SAXS data from the release of HBsAg from SBA-15 in PBS solution, pH 7.4. Changes in the scattering curve can be attributed to the release of HBsAg.

By comparing the time evolution of these two parameters (*i.e.* ρ and *S*_extra_) with those obtained for the samples containing HBsAg, information on the release behaviour was obtained over a time interval of 48 h. These SAXS curves, averaged over 3 h and plotted with intervals of 6 h, are shown in Fig. 5 ▸. We can clearly see that the scattering curves changed as time progressed, and from the analysis of the fitted parameters ρ and *S*_extra_, both normalized to their initial values and were obtained from data averaged over 20 intervals every 30 min, a decrease between 6 and 8 h for both values was observed. This delayed decrease of ρ when compared with the pure SBA-15 L1 sample in solution can be attributed to the PBS salt not being able to diffuse into the mesopores of the SBA-15 L1 sample at the beginning of the interaction, as HBsAg blocks the entrance of the mesopore. However, as soon as HBsAg escaped from the silica the saline solution began to enter the mesopores space. Furthermore, the decrease in *S*_extra_ can be attributed to large aggregates formed inside the dried silica matrix being dissolved in the PBS medium as time evolves. The progression of these two parameters can be interpreted as the release of HBsAg from the SBA-15 structure after 6–8 h of exposure of the vaccine complex (SBA-15 plus HBsAg) in PBS solution, pH 7.4.

The same release time, *i.e.* between 6 and 8 h, observed in the saline solution (*viz* PBS at pH 7.4) was observed for the intestinal test solution (pH 6.8). However, the averaged SAXS curves of the 1:30 sample dissolved in the solution mimicking the gastric fluid conditions (pH 1.2) did not change over time, indicating that HBsAg was not released [Figs. S1(*a*) and 1(*b*) of the supporting information]. Fitting the data averaged for each 30 min interval, as done for previous samples, provided the progression of the parameters ρ and *S*_extra_. We observed that both remained constant over the whole observation frame. Thus, it can be concluded that SBA-15 effectively protected HBsAg at low pH, *i.e.* under gastric acid conditions. This is a very positive property of the delivery vehicle as it prevents the untimely release of the orally administered vaccine under the harsh conditions of the stomach. Furthermore, the SAXS curves of SBA-15 with HBsAg covered with Eudragit in gastric and intestinal solution showed identical behaviour to the former results.

Now we turn to the release profile of HBsAg and dANA in the intestine test solution when encapsulated in laboratory-produced SBA-15 (L2 samples) obtained from the *in situ* SAXS data. For both samples the largest change was at the beginning of the process, indicating that HBsAg was freed from the silica matrix almost immediately. In the case of dANA the details of the release were obtained from the time evolution of the parameters ρ and *S*_extra_ following the steps previously described. The rapid decrease of the parameter ρ can be interpreted as the salt entering the mesopores. Nevertheless, as the dANA might not necessarily block the entrance of the mesopores, due to its small size, one could argue that the diffusion of the salt into the mesopores might not be directly attributed to antigen release. However, turning our attention to the progress of *S*_extra_ we can conclude that its instantaneous increase implies that dANA is quickly released and aggregates in the intestinal test solution. The latter was indeed observed visually when the experiment finished.

Note that the observation of different release profiles of HBsAg – slow release from industrially produced SBA-15 (L1) *versus* the instantaneous process from laboratory-produced L2 – suggests preservation of the chemical and physical properties of the vaccine components observed on a laboratory scale (L2); however, this control during mass production (L1) is an arduous task. These findings also clarify why the immunological response in mice using the silica from L2 samples was significantly larger when compared with conventional oral vaccines. Another important consideration that emerged from these results is that not only SAXS and NAI characterizations have to be performed to check its chemical and structural reproducibility of the OMS, but most importantly its morphology must be investigated to define its influence on the antigen location in the silica meso- and/or macroporosity.

To improve the antibody response the distribution of the antigens inside the silica matrix must be controlled to avoid clustering. Therefore, X-ray and neutron tomography data were collected on the immunogenic complex HBsAg encapsulated in SBA-15 with antigen:silica mass ratios from 1:1 to 1:100 to determine the best mass ratio (*i.e.* the least agglomeration of proteins) is observed. Data obtained by neutron and X-ray attenuation tomography were reconstructed to 16-bit grey-scale tomograms and normalized. The conversion to the absorption coefficient μ was obtained as follows:

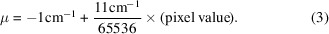

**Figure 6:**
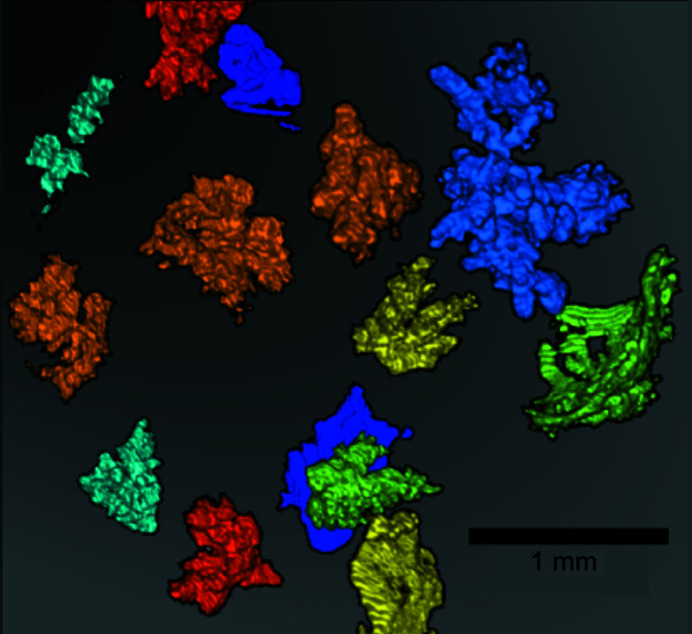
3D visualization of different agglomeration islands of HBsAg in the SBA-15 1:2 sample. Each agglomeration is shown using different colours for clarity.

Areas of high attenuation were observed for both X-ray and neutron measurements, which were visualized at the same locations in the grains of SBA-15, leading us to conclude that HBsAg agglomerated together with the salts in a very non-uniform fashion. The largest agglomerations were observed for the 1:2 ratio and are 3D visualized in Fig. 6 ▸, where colours are used to separate the aggregates. These morphological types of clusters were observed between 1:2 and 1:10 ratios of HBsAg to SBA-15, implying that agglomeration occurred even for samples containing a very small amount of HBsAg. On the other hand, for the 1:1 sample the morphologies registered with X-rays and neutrons were completely different.

**Figure 7:**
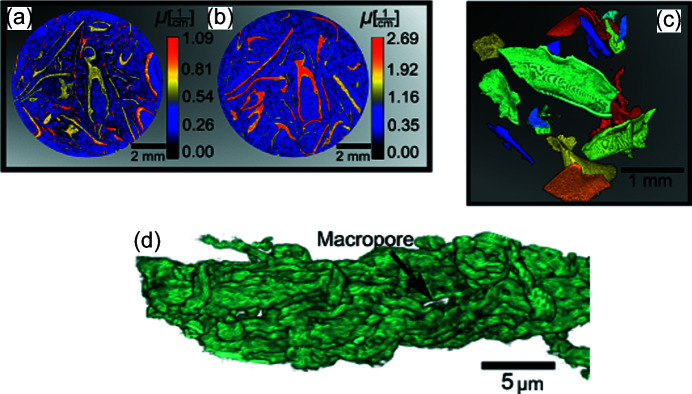
(*a*) X-ray tomogram of SBA-15 1:1 showing clusters with different morphologies than observed for smaller ratios of HBsAg to SBA-15, obtained using the X-ray mCT setup. (*b*) Neutron tomogram of the SBA-15 1:1 sample showing clusters assigned to HBsAg with the same shape observed with X-rays, obtained with the CONRAD instrument. (*c*) 3D visualization of HBsAg aggregates in the SBA-15 1:1 sample. Each aggregate is shown with a different colour. These aggregates have a plate shape which is not observed in any other SBA-15 to HBsAg ratios. (*d*) 3D visualization of the SBA-15 particle using ID16B setup. The silica rods and some larger macropores can be observed, as indicated by the arrow (white region).

In Fig. 7 ▸ both measurements showed long curved structures. By visually observing the powder samples the individual plates could be observed as hard structures resembling crystallization.

To determine the ratio HBsAg:SBA-15 with the minimal amount of agglomeration, an algorithm to index the individual agglomerated objects was created in MATLAB. First a threshold value for μ was defined, so every pixel above this value was considered to be a cluster. The algorithm then labelled every neighbouring pixel as a part of the same cluster. As the agglomerations in the different SBA-15 grains are not touching each other, each agglomeration could be indexed. Information on the agglomeration size and the values of μ were then determined for each cluster. Subsequently, a size filter was implemented to remove structures below a volume threshold as well as random noise, allowing for us to obtain index values of large clusters, their identification and direct comparison between the X-ray and the neutron measurements. From the X-ray and neutron measurements it could be seen that HBsAg agglomerated in large structures in all HBsAg:SBA-15 ratios. Therefore, HBsAg was not uniformly distributed on the length scales accessible by neutron and X-ray attenuation tomography methods. To determine the most efficient HBsAg:SBA-15 ratio, defined by the volume of the agglomeration relative to the amount of encapsulated HBsAg, after filtering the tomograms by cluster size, the relative amount of sample volume containing agglomerated HBsAg was plotted as a function of the SBA-15:HBsAg ratio. The samples with the larger amount of HBsAg contained the largest amount of clustering. Because all samples contained the same amount of PBS, the agglomeration volume observed in SBA-15 plus PBS was subtracted and the amount of agglomeration was scaled by the factor *XP*/100, where *X* is the SBA-15:HBsAg ratio in the sample and *P* is the encapsulation efficiency of HBsAg for the given ratio as defined in our previous work (Scaramuzzi *et al.*, 2016 ▸). It was found that for both neutrons and X-rays the relative agglomeration of HBsAg is smallest in the 1:40 sample and that much of the agglomeration was caused by the salt itself [Figs. S2(*a*) and S2(*b*)].

To verify if HBsAg is present without clustering in other parts of the sample, XPCT and STXM experiments, which have spatial resolution of around 100 nm, were used.

In the XPCT experiments performed at ID16B, the pixel value is proportional to the electron density, thus the reconstructed tomogram of SBA-15 showed that the silica density in the sample was very low. Moreover, macropores, seen as small voids inside the SBA-15 particles, could be easily distinguished. To remove noise and artefacts, the same MATLAB algorithm previously described was used to index all objects with pixel values higher than the threshold, as well as to remove objects too small to be SBA-15 particles. The 3D visualization obtained of an elongated SBA-15 particle is shown in Fig. 7 ▸(*d*). In this visualization the curving subunit rods can be followed along the particle and some of the largest macropores observed. The tomogram of SBA-15 plus PBS showed the agglomeration of the PBS salt in the macropore region. Similarly to the results obtained with CONRAD and X-ray attenuation tomography we observed that salt agglomerated either in the individual SBA-15 particles or on larger grains. These tomograms also showed that not every SBA-15 particle contained agglomerations of PBS, confirming the hypothesis that clusters of PBS are indeed not uniformly distributed in the silica.

Tomograms of 1:2 and 1:10 samples showed that both HBsAg and PBS salt agglomerated in the macropores between the SBA-15 subunits. Similar to the results found for SBA-15 plus PBS, it was observed that not all SBA-15 particles necessarily contained agglomerations. By analysing the surface of the SBA-15 particles we observed only few clusters of HBsAg. However, when these same data were shown with the silica set to be semi-transparent, agglomerated HBsAg could clearly be observed between the silica rods with a preferred parallel direction. These 3D visualizations proved that HBsAg was not present on the surface of the SBA-15 particles, but it was in fact protected in the macropores inside the SBA-15 matrix [Figs. S3(*a*) and S3(*b*)]. Measurements of the 1:1 sample were also performed, showing that the antigen was distributed both inside and on the surface of the SBA-15 particles, in opposition to the localization of HBsAg inside the SBA-15 macropores, for the higher mass ratios.

After analysing the tomograms obtained for SBA-15 plus PBS and for the samples containing HBsAg at various ratios, it was clear that HBsAg agglomerated in the macropores of SBA-15, with a non-uniform distribution. It was also clear that there were SBA-15 particles that contained no distinguishable agglomerations, as well as many macropores that did not contain agglomerated HBsAg. This does not mean that there is no HBsAg in these areas, but merely that the antigen can also be protected in the macropores without agglomerating. To determine if this was indeed the case, the distribution of pixel values for silica for each sample was compared. In this case, a large number of pixels with high values in non-agglomerated regions of the silica with HBsAg was attributed to the presence of non-agglomerated HBsAg. Therefore a probability density function (PDF) was obtained for the pixel values of the non-agglomerated silica for all tomograms. Pixel values above 110 were attributed to silica and values above 150 were considered to be agglomeration. All the HBsAg agglomerations were indexed and removed from the PDF together with pixels neighbouring the agglomerations to avoid pixel values slightly smaller than 150, probably also containing HBsAg due to their proximity. Pure SBA-15 has a lower probability of having higher pixel values than the samples containing PBS and HBsAg. The full analysis of the PDF from the various antigen:silica mass ratios revealed the number of high pixel values of the 1:40 sample was indistinguishable from SBA-15 plus PBS. However, it cannot yet be established whether the 1:40 sample contained non-agglomerated HBsAg, even though it was the ratio with the lowest agglomeration yield [Figs. S4(*a*) and S4(*b*)].

In addition, a tomogram of SBA-15 with dANA showed aggregation of PBS and possible dANA in the macropores of SBA-15. For SBA-15 plus PBS the calculated agglomeration percentage was 6 ± 1% and for the 1:10 sample with dANA it was 5 ± 1%. No additional agglomeration was observed when dANA was introduced in SBA-15, thus indicating that this antigen did not agglomerate inside the silica, at least not for the mass ratio 1:10 [Figs. S5(*a*), S5(*b*) and S5(*c*)]. This is in agreement with the SAXS and TEM results, which showed the encapsulation of dANA inside the mesopores.

Finally, STXM and NEXAFS data were collected to give insight into the distribution of HBsAg and dANA in SBA-15 particles, from sources L1 and L2, respectively. STXM images of pristine SBA-15 taken at 390 eV (*i.e.* above the carbon edge 282 eV) showed no detectable carbon inside the particles, as expected; whereas images of an SBA-15 plus PBS presented a higher attenuation, which can be attributed to either the presence of salt or the thickness of the particle. On the other hand, using this same energy STXM image of the 1:10 (HBsAg) sample showed two very dark areas due to very high attenuation and the agglomerations of HBsAg observed with XPCT. A NEXAFS linescan broad spectrum taken inside the particle was identified as a result of C—N and C—H bonds in HBsAg that are convoluted non-trivially with each other (Stöhr, 1992 ▸). For the NEXAFS spectrum obtained at the centre of this particle a clear carbon absorption edge as well as peaks attributed to the many carbon bonds of HBsAg were registered. From these results it could be concluded that particles with and without HBsAg agglomerations are present, confirming the interpretations of the XPCT analysis.

**Figure 8:**
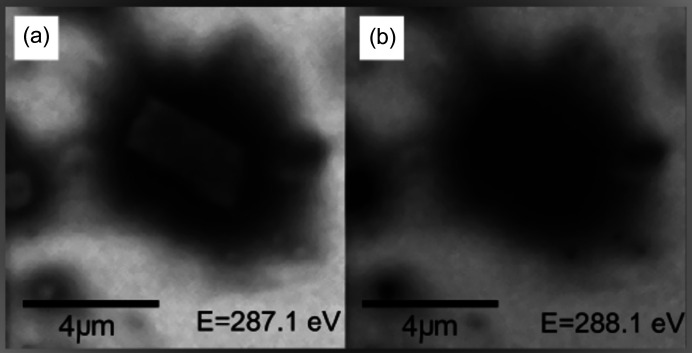
(*a*) STXM image of a particle from the 1:1 sample with 287.1 eV photon energy, just above the C=O bond peak showing differences of attenuation between the contamination around the particle and HBsAg. (*b*) STXM images of a particle from the 1:10 sample with 288.1 eV photon energy in the range attributed to C—H and C—N bonds.

Figs. 8 ▸(*a*) and 8(*b*) show STXM images obtained at 287.1 and 288.1 eV, just above the C=O peak and in the broadening caused by C—H and C—N bonds. For 287.1 eV the C=O bonds were turned ‘on’ in the image region where we observed contaminations around the particle, which could easily be distinguished from the particle itself. This contamination attenuated the X-ray beam more, as a result of a larger number of C=O bonds. However, for 288.1 eV when the C—H and C—N bonds were turned ‘on’, similar attenuation was shown for the particle and for the surrounding contamination. This data confirmed the distinction between the carbon signal obtained from HBsAg inside the particle and that obtained outside the particle from air contamination.

Linescan spectra taken at the centre of the particles for all SBA-15:HBsAg ratios were measured in particles with no agglomerations. The antigen was visibly present in non-agglomerated form. This result further confirmed that the 1:40 sample contained non-agglomerated HBsAg, complementing the missing information from the XPCT analysis.

The focus is now shifted to SBA-15 with encapsulated dANA where no agglomerations were located in any of the particles scanned during the experiment, which is in agreement with the observations of XPCT and further confirms that dANA does not aggregate inside SBA-15. The NEXAFS spectra indicated the presence of carbon inside the particles when dANA was encapsulated in the silica, corroborating the presence of the antigen inside the mesopores of SBA-15, as also shown by TEM data (Rasmussen *et al.*, 2021 ▸).

**Figure 9:**
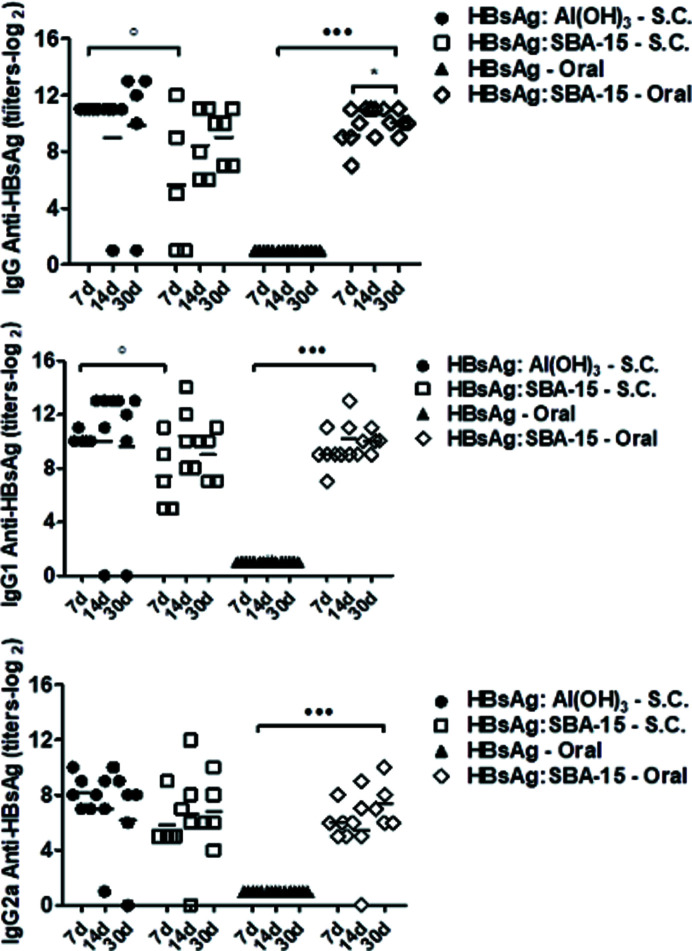
Anti-HBsAg serum IgG and subclass titers detected by ELISA in BALB/c mice immunized with HBsAg, encapsulated/adsorbed in mesoporous SBA-15 or adsorbed in Al(OH)_3_ by subcutaneous (SC) or oral (OR) routes. The titers were detected 7, 14 and 30 days after the booster. A group of animals immunized with rHBsAg was used as a reference for the unpaired Student t test analysis, *p < 0.05; ⋯ *p* < 0.001. Antibody titers at days 7, 14 and 30 after the booster were also used as a reference for the unpaired Student t test analysis, °*p* < 0.05 [reproduced from the work by Scaramuzzi *et al.*, 2011 ▸)].

Finally, the antibody titers for oral (OR) and subcutaneous (SC) administration of these vaccine complexes (L2 type) are shown in Figs. 9 ▸ and 10 ▸ for HBsAg and dANA, respectively. Both were also compared with the human certified adjuvant Al(OH)_3_. These results proved the efficiency of this new formulation for oral vaccines, since a significant level of antibodies (in logarithmic scale) of oral immunization with silica are comparable with subcutaneous via and higher than those administered with Al(OH)_3_ (Scaramuzzi *et al.*, 2016 ▸; Rasmussen *et al.*, 2021 ▸).

## 4. Conclusions

Our results emphasize how crystallography is key in describing morphology, determining protein aggregation and helping in technological development of SBA-15 as a carrier for oral vaccines. Here by employing a variety of state-of-the-art solid-state techniques, we determined the best protocol to develop an efficient oral vaccine without minor protein clustering.

Firstly, SAXS and TEM confirmed that the SBA-15 samples prepared on either an industrial scale (labelled L1) or a laboratory scale (labelled as L2) had the expected hexagonal-ordered mesoporous structure, but SEM images showed different morphologies of the subunits. L1 samples were shaped as curved rods with lengths around 2 µm, whereas L2 contained a mixture of rods and beads. These different morphologies were detected even though L1 and L2 were synthesized according to similar chemical recipe and process. This crucial information clearly shows that slight changes in synthesis parameters strongly affect the SBA-15 morphology and consequently the immunogenic response; L1 did not induce the desired immunological response of an oral vaccine made with HBsAg and tested in mice, whereas another sample produced at laboratory scale (Scaramuzzi *et al.*, 2016 ▸) produced a remarkable amount of antibodies. Therefore, a compromise among scalability and structural integrity is essential for the efficiency of SBA-15 as vaccine adjuvant. The role of the OMS morphology in the effectiveness of oral vaccine formulation is still an open question. Additionally, TEM detected that the dANA entered the 10 nm mesopores of SBA-15, while HBsAg remained in the silica macroporosity. This was expected as this virus-like particle was too large to be inside the mesopores.

By combining X-ray and neutron attenuation tomography, using the same pixel size of 6.37 µm, we showed that the PBS salt and HBsAg agglomerated together in the powder grains of SBA-15. Similar results were obtained using SEM and EDS analysis. Agglomerations of the same morphology were observed for all HBsAg:SBA-15 ratios in the tomograms, except for the ratio with the largest antigen ratio (1:1), where crystallite-like structures are seen. By normalizing the volume of the agglomerations in SBA-15 to the amount of encapsulated HBsAg, we observed that the ratio for which HBsAg agglomerated the least is 1:40. This is expected to be the optimal value for vaccination, as agglomerated antigen is expected to negatively influence the immunological response.

Additionally, phase-contrast tomography showed that the PBS salt and HBsAg agglomerated in the macropores (>50 nm) created between the silica subunits in the large particles of SBA-15. However we observed that not all SBA-15 particles contained clusters of the antigen. These measurements further confirmed that the ratio 1:40 is the one where the least agglomeration of HBsAg was observed. In fact, for this ratio the amount of agglomeration caused by the PBS salt and that caused by the addition of the antigen are indistinguishable. From the phase-contrast tomography data it was also concluded that the SBA-15 particle did not increase in size when HBsAg was encapsulated. This is an important result, as the particle size must remain stable for use in real vaccines. Phase contrast tomography measurements on the small-scale-produced SBA-15 (L2) showed that although PBS agglomerated inside, the encapsulation of dANA did not induce an extra signal, in perfect agreement with the fact that dANA was trapped inside the 10 nm mesopores. From statistical analysis of the pixel values of silica without agglomeration of PBS plus HBsAg we showed that HBsAg was also distributed in the SBA-15 particles with and without agglomeration. This was further confirmed using STXM and NEXAFS around the carbon *K*-edge, where the presence of the antigen could be detected in all measured SBA-15 particles. Interestingly, by employing STXM and NEXAFS non-agglomerated dANA was detected at all measured SBA-15 particles. Furthermore, to determine if the agglomeration of HBsAg in SBA-15 was caused by the antigen aggregating in solution even before encapsulation into SBA-15, SAXS measurements were performed. From the SAXS data the shape of HBsAg in solution was modelled as an unclosed sphere with a maximal internal distance of 32 ± 4 nm, confirming that HBsAg is too large to enter the mesopores of SBA-15 (Rasmussen *et al.*, 2021 ▸). The shape of HBsAg was shown to be stable while varying the experimental conditions: temperature, pH of the buffer and storage temperature of the sample. By varying the pH of the buffer, it was shown that the particle has the smallest radius of gyration and molecular weight at pH 7:4 showing that no decrease of agglomeration in SBA-15 can be expected by changing the pH of HBsAg (Lopes *et al.*, 2019 ▸; Rasmussen, 2017 ▸). SAXS modelling of HBsAg is consistent with the TEM and DLS measurements. The latter showed that 3–10% of the HBsAg volume in solution formed larger aggregates, which could not be detected using SAXS. The amount of aggregation did not depend significantly on the pH (in the range 5.5 to 8.5) of the buffer confirming that no decrease of agglomeration of HBsAg in SBA-15 can be expected by changing the pH from 7.4. From DLS data, we also observed that the HBsAg samples kept for four days at −20°C started to aggregate quite a lot, whereas samples kept at room temperature for four days did not aggregate more than those kept at 4°C. SAXS and DLS were also used to model dANA as an elongated particle with a length of 10 nm and a diameter of 3–4 nm in the non-elongated direction, confirming that dANA could enter the 10 nm mesopores of SBA-15. Additionally DLS showed that no larger aggregation of dANA occurred (Rasmussen *et al.*, 2021 ▸; Rasmussen, 2017 ▸). SBA-15 powder samples with HBsAg and dANA were measured with SAXS and fitted to the model described in Equation (1). The fit results indicated that HBsAg was attached to the mesopores, blocking their entrance. This analysis also showed that the agglomeration of HBsAg occurred in both synthesized SBA-15 matrices L1 and L2. SAXS measurements on L2 also confirmed that dANA entered the mesopores without agglomerating. This protection was also in agreement with TGA data, which showed an increase of dANA degradation temperature when encapsulated inside SBA-15 (Rasmussen *et al.*, 2021 ▸).

Finally, *in situ* SAXS experiments showed that for the industrially produced SBA-15 (L1) HBsAg is protected from gastric acid test solution. On the other hand, release in intestinal fluid is quite slow compared with the immediate release observed for the SBA-15 synthesized on a small scale (L2). Similar fast-release behaviour was observed for encapsulated dANA. The delayed-release initiation from industrially produced SBA-15 can be the reason why the required immunological response was not observed in the oral vaccine produced using L1. These results demonstrated the necessity to consider morphological characteristics as an important parameter to be monitored in order to prepare efficient oral vaccines.

## Supplementary Material

Supporting figures. DOI: 10.1107/S205225252101071X/lt5043sup1.pdf

## Figures and Tables

**Figure 10:**
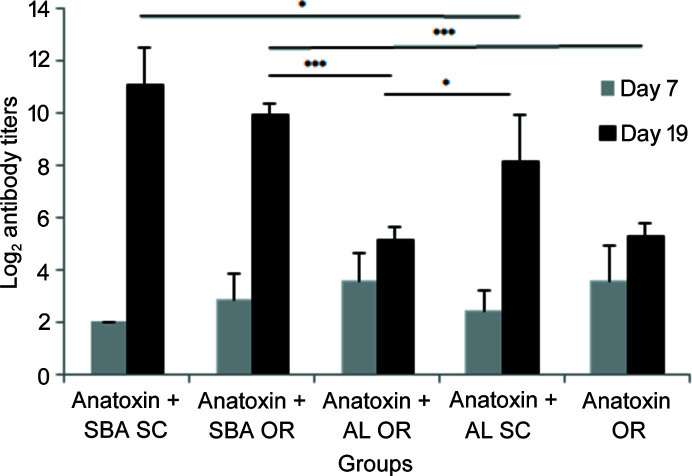
Anti-diphtheria serum IgG titers detected by ELISA in BALB/c mice immunized with dANA encapsulated/adsorbed in mesoporous SBA-15 or adsorbed in Al(OH)_3_ by subcutaneous (SC) or oral (OR) routes. The titers were detected 7 (grey columns) and 19 (black columns) days after immunization. The statistical analyses were performed with the one-way ANOVA test (*P < 0.05; ***P < 0.005) [reproduced from the work by Rasmussen *et al.* (2021 ▸)].

**Table 1:** Description of the samples measured during *in situ* SAXS experiments The composition of the solutions followed the description given by the US Pharmacopoeia, which can be found at http://www.pharmacopeia.cn/v29240/usp29nf24s0_ris1s126.html: PBS (250 ml water, 2.0 g NaCl, 0.05 g KCl, 0.35 g Na_2_HPO_4,_ 0.06 g KH_2_PO_4_), intestine (250 ml water, 19 ml NaOH 0.2M, 1.7 g KH_2_PO_4_) and gastric (250 ml water, 1.75 ml HCl, 0.5 g NaCl). L1 is the industrial-prototype OMS sample and L2 is the small-scale OMS sample

Sample	Solution (OMS sample)
SBA-15	PBS (L1 and L2)
1:30(HBsAg)	PBS (L1 and L2), intestine (L1 and L2), gastric (L1)
1:30(HBsAg) plus Eudragit	Intestine (L1), gastric (L1)
1:10(dANA)	Intestine (L2)

**Table 2:** Textural parameters of pure SBA-15 (the uncertainty is related to slight variations of the synthesis process), SBA-15 encapsulated with only PBS (the large uncertainty is due to different amounts of PBS) and SBA-15 encapsulated with PBS plus the antigens inside the silica (antigen:SBA-15 plus PBS)

Sample	BET surface area (m^2^ g^−1^)	Pore volume (cm^3^ g^−1^)
SBA-15	700 ± 30	1.61 ± 0.10
0: SBA-15 plus PBS 1:5	170 ± 70	0.66 ± 0.39
(HBsAg) 1:10	136	0.61
(HBsAg) 1:20	194	0.87
(HBsAg) 1:40	166	0.72
(HBsAg) 1:5	188	0.83
(dANA) 1:10	1.3	0.0051
(dANA) 1:25	15	0.044
(dANA) 1:50	98	0.27
(dANA)	65	0.18

**Table 3:** Table of fitting parameters used in the *I*(*q*) equation

Parameter	Description
*S*_1_	Scale factor describing the mesopore contribution
*S*_micro_	Scale factor describing the micropore contribution
*a*	Lattice spacing describing the hexagonal lattice
*D*	Domain size of the hexagonal ordered mesopores
*ν*	Peak-shape parameter
σ_a_	Disorder parameter, describing the disorder of the hexagonal lattice
*R*_in_	Inner radius of the cylinders describing the mesopores
*R*_out_	Outer radius of the cylinders describing the mesopores
*L*	Length of the cylinders describing the mesopores
ρ	Ratio between the outer an inner electron density of the mesopores
σ_int_	Smearing parameter for the outer cylinder
σ_*R*_/*R*	Relative polydispersity of the mesopores radii
*R*_g_	Radius of gyration of the micropores
*S*_extra_	Scale factor for the contribution arising from unspecific agglomerates
*S*_*qb*−4_	Scale factor for the *q* ^−4^-dependent contribution, describing the Porod contribution of the mesoporous domain
Back	Constant Background
