# Supplementary material for: Using crystallography tools to improve vaccine formulations
Source: IUCrJ. 2021 Nov 25;9(Pt 1):11–20. doi: 10.1107/S205225252101071X (PMC8733884; doi:10.1107/S205225252101071X)
Supplement: Supplementary file 1 [file m-09-00011-sup1.pdf]

IUCrJ

Volume 9 (2022)

Supporting information for article:

Using crystallography tools to improve vaccine formulations

Márcia Carvalho de Abreu Fantini, Cristiano Luis Pinto Oliveira, José Luiz de Souza Lopes, Tereza da Silva Martins, Milena Apetito Akamatsu, Aryene Góes Trezena, Milene Tino-De- Franco, Viviane Fongaro Botosso, Osvaldo Augusto Brazil Esteves Sant'Anna, Nikolay Kardjilov, Martin Kjaerulf Rasmussen and Heloísa Nunes Bordallo

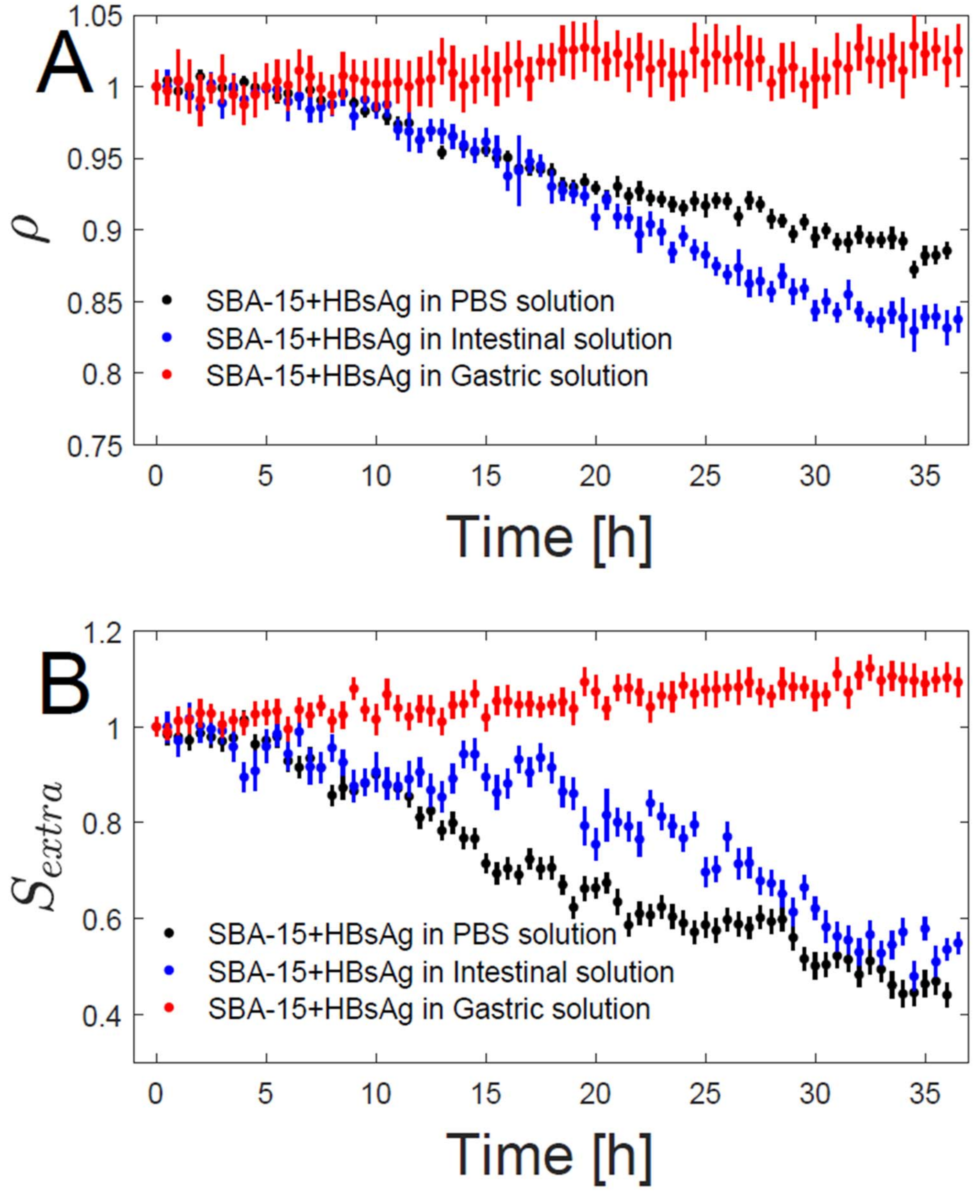

Figure S1. Fit parameters as a function of time describing the release of HBsAg from SBA-15 experiments in PBS, intestine and gastric acid test solutions, pH 7.4, 6.8, 1.2, respectively. (A) ρ related to the electron density in the mesopores normalized to its initial value. (B) S_{extra} proportional to the amount of agglomeration also normalized to its initial value.

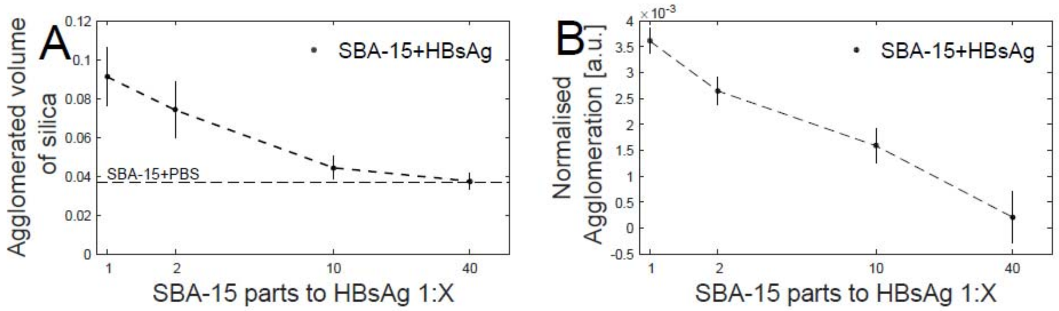

Figure S2. (A) Fraction of agglomerated SBA-15 volume. (B) Agglomeration amount normalized to encapsulated HBsAg mass by the approach described in the text.

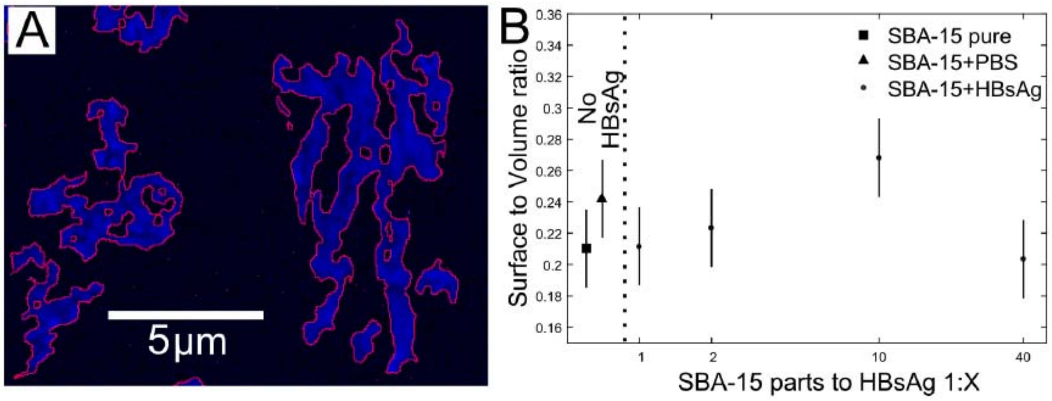

Figure S3. (A) Part of SBA-15 tomogram where the pixels counted as surface pixels are marked red. (B) Surface to volume ratios as a function of SBA-15 to HBsAg ratio. The particles size does not increase as the HBsAg is encapsulated. The numerical values of the surface to volume ratio have no significance as they are totally dependent on the binning of the pixels in the tomograms.

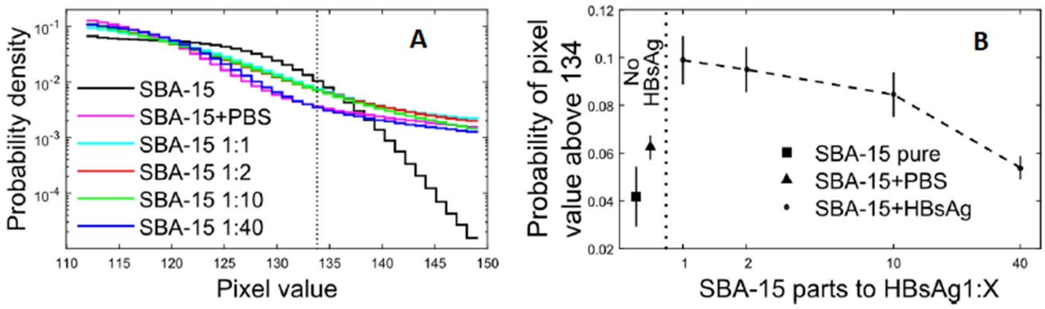

Figure S4. (A) Probability density function (PDF) of pixel values for all samples. (B) Values obtained from integrating the probability density function (PDF) between the values 134 to 150.

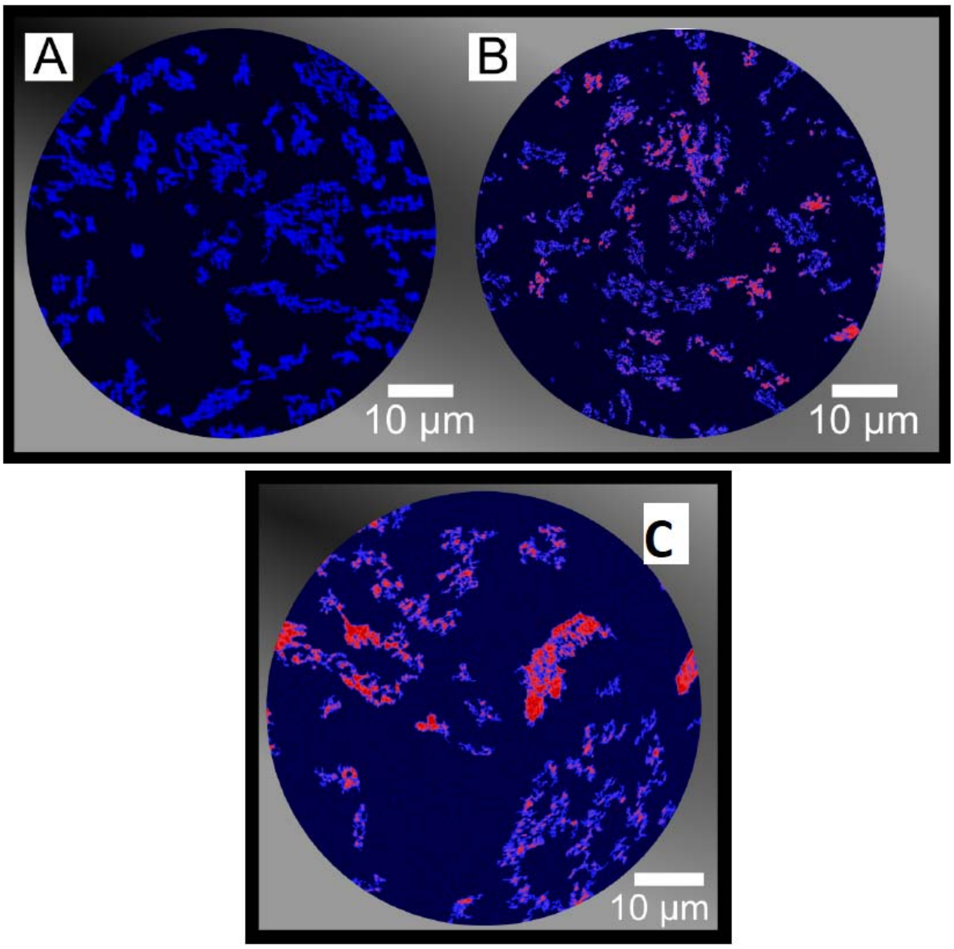

Figure S5. (A) Tomogram of L2 sample prepared with pure SBA-15. (B) Tomogram of the same sample plus PBS. (C) Tomogram of the same SBA-15 (L2) plus PBS plus dANA (1:10).
